# Experiment-based calibration: Inference and decision-making

**DOI:** 10.3758/s13428-026-03042-9

**Published:** 2026-06-04

**Authors:** Federico Mancinelli, Dominik R. Bach

**Affiliations:** 1 Centre for Artificial Intelligence and Neuroscience, Transdisciplinary Research Area Life and Health, University of Bonn, Bonn, Germany; 2 Institute of Computer Science, University of Bonn, Bonn, Germany; 3 University Hospital Bonn, Institute of Experimental Epileptology and Cognition Research, University of Bonn, Bonn, Germany; 4 University Hospital Bonn, Department of Psychiatry and Psychotherapy, University of Bonn, Bonn, Germany; 5 Department of Imaging Neuroscience, Queen Square Institute of Neurology, University College London, London, UK

**Keywords:** Calibration, Retrodictive validity, Metrology, Psychometrics, Measurement accuracy, Measurement uncertainty

## Abstract

Experiment-based calibration is an emerging approach for measurement validation in the behavioural sciences. It allows comparing multiple measurement methods by how well they reproduce a known experimental manipulation, providing insight into their measurement accuracy. Calibration entails questions unparalleled in classical validation approaches. The first is about inference: when should we conclude that one measurement method is truly more accurate than another? The second is about decisions: when should we decide that a method merits the investment of changing a measurement system? In this note, we review these questions in the context of the statistical challenges that arise in a calibration process: a potentially large and a priori unknown number of measurement methods; a requirement to integrate evidence across multiple calibration samples; and a possibility that some methods may not be available for all samples. We show that Bayesian meta-analytic model comparison is a suitable framework for inference in calibration, and propose a decision-theoretic approach to calculate immediate economic gain garnered through reduced sample sizes. In order to overcome the practical hurdles associated with the analysis of calibration experiments, we present *CalibR*, an R package for calibration inference.

## Introduction

Empirical research in many areas of behavioral science necessitates the measurement of latent variables (also termed, with slightly different nuances, “attributes” or “constructs”). This raises questions of validity (AERA, 2014): does the measurement capture the latent variable, and if so, how well? To answer such questions, measurement validation includes the assessment of quantitative metrics (AERA, 2014). Classical validation theory, grounded in the study of individual differences (Cronbach, 1957), focuses on convergent/discriminant validity and reliability (McDonald, 2013). While these metrics are successfully applied to stable enduring traits (Widaman, 1985), they can be more difficult to assess outside this core domain due to conceptual and practical constraints. Conceptually, classical validity metrics are only meaningful when there is sufficient between-person variability in a latent variable (Bach, 2023). In the study of general treatment effects, such as in experimental psychology, this is often not the case (Hedge, Powell, & Sumner, 2018). For example, extraversion is a latent variable with typically large between-person variability, making it amenable to classical validation. In contrast, situational anxiety in an emotion-inducing experiment may have low between-person variability if the experimental treatment is highly effective. Practically, convergent validity and reliability can become uninterpretable due to method effects; that is, systematic influences introduced by the measurement method, such as scoring rules or response modalities (Bach, 2023). For example, people’s measured scores in two tests using language might be correlated not because the tests measure the same thing, but because they both involve (stable) linguistic skills. This necessitates finding unrelated methods to assess convergent validity and unrelated latent variables to assess discriminant validity. In fact, this provides a high bar for validation in many fields (Bach, Rigdon, & Sarstedt, 2025), so that validity assessment is in practice often incomplete (Zumbo & Chan, 2014) and in these cases, hard to interpret.

Experiment-based calibration provides an alternative validation framework for latent variables that are malleable by experimental manipulation (Bach, Melinscak, Fleming, & Voelkle, 2020). Experiment-based calibration is based on a treatment that is known to change the values of a latent variable. The correlation of measured values with the predicted *standard* values of the latent variable in this experiment constitutes a type of criterion validity and has been termed *retrodictive validity* (Bach & Melinscak, 2020). While the numerical values of retrodictive validity are not meaningful for one measurement method on its own, they provide a basis for comparing several measurement methods for the same latent variable. Under fairly general assumptions, higher retrodictive validity guarantees higher measurement accuracy of a method (Bach, 2025; Bach et al., 2020).

At this point, it is useful to consider what constitutes a “measurement method”. As an example, a questionnaire contains a number of items which participants answer – thus generating “observables”. These observables are then pre-processed to derive a measured score of the latent attribute, for example, by forming a sum score – we term this “transformation”. In our terminology, a measurement comprises the entire process of generating a measured score, including observation and any data transformations. There are cases where observation and transformation cannot be separated, such as when a standard questionnaire has a canonical sum score. On the other hand, even for questionnaires, there are sometimes different scoring methods, and experimental observations – from reaction times to psychophysiological time series – can be pre-processed in a myriad of ways (Simmons, Nelson, & Simonsohn, 2011; Steegen, Tuerlinckx, Gelman, & Vanpaemel, 2016; Silberzahn et al., 2018; Botvinik-Nezer et al., 2020). All of these different data transformations define separate measurement methods, which can be compared in a calibration experiment.

While classical measurement validation is often focused on jointly validating a small set of measurement methods, experiment-based calibration inherently mandates competition between methods. In practical applications, it has turned out that very similar measurement methods can be usefully compared and ranked. For example, pre-processing strategies for time-series data in psychophysiology often include filtering. In a calibration setting, a large range of filter cut-off frequencies can be evaluated to select the optimal one (e.g., Korn, Staib, Tzovara, Castegnetti, & Bach, 2017; Castegnetti, Tzovara, Staib, Gerster, & Bach, 2017; Paulus, Castegnetti, & Bach, 2016; Staib, Castegnetti, & Bach, 2015; Khemka, Tzovara, Gerster, Quednow, & Bach, 2017). Another example is the many different workflows for reaction-time outlier detection (Simmons et al., 2011) that could be compared in a calibration experiment.

This emphasis on comparison presents challenges unforeseen within the framework of classical validity theory. In this note, we outline these challenges and relate them to the common probabilistic frameworks of statistical hypothesis testing, Bayesian parameter estimation, Bayesian model comparison, and decision theory. We propose a method for analyzing calibration experiments, which we implement in the R package *CalibR*.

## Calibration in practical settings

After conducting a calibration experiment, research practitioners will often have two questions. The first is: which is the best among all the tested measurement methods in the sample, and how certain can we be that it truly is the best method in the population? Unless this is the best method they already use, their second question might be: how much better is this method compared to the one I am currently using, and is that difference worth the investment to change my current method?

In order to be unbiased and precise, calibration should ideally be a large-scale, multi-laboratory effort. In a recent example, a 25-laboratory collaboration proposed a consensus design for a calibration experiment for human fear conditioning (Bach et al., 2023), which is currently being implemented with a large target sample size. Such investment is only viable if many different measurement methods can be compared and if the source data are available for future method development and comparison. Thus, the first challenge in answering the aforementioned questions is to account for a large and a priori unknown number of comparisons between methods.

Next, the results of a calibration experiment – the retrodictive validity scores – are expressed as Pearson correlation coefficients. For each measurement method, this correlation coefficient depends on measurement accuracy – which is what we are interested in – and on experimental aberration. The latter quantity expresses how close the true scores of the latent variable in the calibration experiment are to the standard scores, or in other words, how effective the experimental manipulation was. This quantity is, of course, unknown and will likely depend on the experimental procedure as well as on sample characteristics. As such, for each tested measurement method, the numerical retrodictive validity scores might differ between two different calibration experiments – even if the ranking of measurement methods is exactly the same for each of the experiments. In particular, it has been suggested that calibration experiments can be iteratively improved (for example, to allow smaller sample sizes), and in this case, retrodictive validity scores will become systematically larger over time (Bach et al., 2025). Nonetheless, to the extent that two calibration experiments have the same validity conditions (sample characteristics, effectiveness of the experimental manipulation, etc.), it would be desirable to integrate information from two experiments. Thus, the second challenge is to combine the rankings of measurement methods from different calibration experiments, with differing retrodictive validity scores.

Finally, not all calibration experiments (or all datasets in a calibration experiment) will include the same set of measurements. For example, in a multi-lab experiment, some sites might lack a certain measurement system (such as an eyetracker); or a calibration experiment might include several previously validated observables together with a novel one. While the novel methods can then only be evaluated on the new data set, it would nevertheless be desirable to use all available data to evaluate the previously known measurement methods. Thus, the third challenge is to deal with missing information for some methods but not others. In the next section, we evaluate common statistical frameworks according to their suitability for this problem setting.

## Inference in a calibration setting

### Frequentist statistics

In statistical hypothesis testing (also termed null hypothesis significance testing), one evaluates how incompatible the observed data are with a specified null model, using a test statistic and its sampling distribution under that model. Applying this to our case, we would like to know if a difference between two measurement methods in their retrodictive validity scores is due to random variability in the measured scores (i.e., the null hypothesis), or if it reflects a systematic pattern in the underlying population (i.e., the alternative hypothesis). Because we draw a finite sample from the population, we can never be sure about this, so we constrain the false positive rate, i.e., the probability of obtaining a similar or a more extreme difference if the data were generated under the null hypothesis. When we compare multiple pairs of methods, each statistical test has a false-positive rate; thus the overall false-positive rate is defined by the number of statistical tests and their stochastic dependence. In calibration, the number of possible statistical tests scales with the squared number of methods, which by itself can be problematic: with a large number of methods, very large samples will be required to pass any significance threshold. The deeper problem, however, is the unknown number of tests: one can always invent new data transformations post hoc, and testing these would then have a post hoc influence on the significance level of tests performed earlier. Statistical hypothesis testing also appears as an unsuitable framework for epistemic reasons: the goal of calibration is frequently not to pitch two specific hypotheses against each other, but rather to compare and rank a large number of methods. Finally, it is difficult to see how statistical hypothesis testing could integrate method rankings across multiple calibration experiments.

### Bayesian parameter estimation

Bayesian parameter estimation treats parameter values as random variables and combines prior knowledge with the likelihood of the data to derive a posterior distribution of parameter values. Thus, one could regard retrodictive validity scores as parameters, estimate their distribution, and specify “credible intervals”, e.g., intervals in which the true values are to be found with 95% posterior probability. Combined with a suitable loss function for making the wrong choice of measurement method, this approach would allow an easy solution to the problem of parameter estimation and decision between methods. Since the goal is not to constrain the false positive rate, but rather to minimize the loss associated with a wrong decision, this framework bypasses the challenge of multiple and a priori unknown number of methods. However, when we integrate method rankings from two or more calibration experiments, performed with different procedures, and thus yielding globally different retrodictive validity scores, their posterior parameter distributions are truly different between experiments. In fact, we do not seek to combine these parameter distributions, but only their rankings. Thus, Bayesian parameter estimation, applied to the retrodictive validity scores, appears to be an unsuitable framework for integrating results across multiple calibration experiments.

### Bayesian model selection

The goal of model selection is to identify the best out of a set of models according to some criterion. The meaning of “model” in model selection can be broad. A widespread application of the model selection framework is the identification of a data-generating model, such as to explain some empirical data. A measurement method can be thought of as a two-step process: an observation step, in which observables are generated from a latent variable, and a transformation step, in which the observable-generating process is “undone” or inverted, to yield an estimate of the latent variable (Bach & Friston, 2013). This view is, for example, formalized in structural equation models (Bollen, 1989), or psychophysiological models (Bach et al., 2018). Thus, in the calibration setting, we can think of measurement methods as “models” that seek to retrodict the standard values. As a criterion to decide between any two models, Bayesian model selection draws on the evidence in favor of one as opposed to another model – this ratio is termed “Bayes factor”.

Bayes factors possess several theoretical properties that make them appealing for calibration. First, their primary function is epistemic, as they aim to quantify relative evidence between models rather than constrain false positive rates. Consequently, Bayes factors provide a direct and interpretable comparison between any two models, independent of the presence or absence of other models. Secondly, Bayes factors integrate over parameter values, making them independent of specific parameter estimates. This allows comparisons even when parameter estimates differ across studies, and thus makes it easy to integrate method rankings across studies. A caveat arises when measurement methods are missing for some of the studies. Bayes factors are transitive (i.e., if model $m_1$ is 10 times more supported than $m_2$, and $m_2$ is ten times more supported than $m_3$, then $m_1$ is 100 times more supported than $m_3$), which potentially allows comparing methods that have not been evaluated within the same studies. However, this property holds only when the comparisons are based on the same data. If Bayes factors are computed on different datasets, they are conditioned on different observations, and thus they should only be combined under an assumption of data homogeneity. In calibration research, this assumption may sometimes be reasonable, for example, when studies use the same calibration procedure and draw from the same target population. Even so, partial method overlap remains an important limitation without a simple general remedy, with the most conservative solution being to restrict comparisons to methods evaluated on the same underlying data.

With this caveat in mind, Bayesian model selection still addresses the practical challenges outlined in Section “Calibration in practical settings”. In the next section, we outline how LogBF can be computed in calibration experiments.

### Computing Bayes factors in *CalibR*

In a calibration experiment, the retrodiction model amounts to a linear regression in which the standard scores serve as the dependent variables, and the measured scores are used as the predictors (independent variables). If there are no repeated measures per condition and per subject, the retrodiction model simplifies to $s \sim 1 + y$ (or equivalently $s \sim y$ if the observations are centered). Here, *s* denotes the standard scores, *y* represents the measured scores, and the notation 1 indicates the intercept, following standard R model syntax. In the presence of repeated measures, *CalibR* uses the deviation from each participant’s mean as the predictor. In terms of the residual sum of squares, this is equivalent to the inclusion of participant-level intercepts.

In order to combine model evidence across studies, it might seem intuitive to multiply their Bayes factors. However, the marginal likelihood for a combined dataset does not equal the simple product of marginal likelihoods from individual datasets because subsequent data should be evaluated conditionally, taking into account previously observed data (Rouder & Morey, 2011). A more principled approach is to integrate evidence by combining study-level test statistics and subsequently mapping these to Bayesian evidence. Throughout, Bayes factors are computed under a standard Gaussian linear regression model with a *g*-prior on the regression coefficients, which yields a closed-form relationship between test statistics and marginal likelihood ratios (Nikolakopoulos & Ntzoufras, 2021).

Thus, *CalibR* computes the *t*-statistic of the retrodiction model for each experiment. These are then combined using inverse variance weighting, resulting in a summary test statistic $\tilde{T}$ across studies. This corresponds to a fixed-effect evidence synthesis under the assumption of a common retrodictive effect across calibration datasets. In the case where the *t*-statistics and sample sizes are fully available for each study, the weights are computed according to the variance of each study’s statistic, specifically:

1 $$\begin{aligned} w_k = \sqrt{\frac{v_k^{-1}}{\sum _{k=1}^K v_k^{-1}}} \end{aligned}$$

Here, $w_k$ denotes the weight assigned to study *k*, $v_k$ is the variance of the *t*-statistic $T_k$ for study *k*, and *K* represents the total number of studies included in the meta-analysis. The combined statistic $\tilde{T}$ is computed as:

2 $$\begin{aligned} \tilde{T} = \sum _{k=1}^K w_k T_k \end{aligned}$$

Here, $T_k$ is the signed *t*-statistic from study *k*, with the direction of the effect encoded in the statistic itself. This formulation aggregates evidence across studies, accounting for effect directionality. The combined statistic $\tilde{T}$ is then converted to a Bayes factor analytically using the functional relationship between the *t*-statistic and the Bayes factor under the *g*-prior (this is a prior distribution for regression coefficients, where the covariance structure is scaled by a single parameter *g*). Specifically, the combined $\tilde{T}$ is plugged into

3 $$\begin{aligned} 2 \log \textrm{BF}_{10} = (N-2) \log (1+g) - (N-1) \log \left[ 1 + g \left( \frac{\tilde{T}^2}{N-2} + 1\right) ^{-1}\right] \end{aligned}$$

where *N* is the total sample size and *g* is the *g*-prior parameter (usually set to *N*), yielding the overall meta-analytic Bayes factor. This procedure ensures that the combined Bayes factor accurately reflects the evidence across studies.

Bayes factors compare the evidence for model $m_1$ relative to model $m_2$. To anticipate a scenario in which not every method is included in every calibration experiment, *CalibR* uses a null model that includes only the intercept and has no information on the measured scores, thus providing the smallest possible retrodictive validity. Bayes factors are computed to compare each model to this common null model. This construction permits joint interpretation of Bayes factors obtained from models fitted to partially non-overlapping datasets, subject to assumptions of sufficient data homogeneity.

The interpretation of Bayes factors from partly non-overlapping datasets is not trivial. To address this, *CalibR* highlights how much data overlap exists between any two methods being compared, and also reports Bayes factors using only completely overlapping data.

## Decision-making in a calibration setting

We now turn to the practical question of decision-making: if calibration suggests that method $m_2$ is more accurate than the currently used method $m_1$, is the gain worth the switch cost – such as new equipment, staff training, or analysis pipeline changes? To address this, we focus on *quantifiable gains*, specifically reductions in required sample size due to improved measurement accuracy, though it is worth noting that non-quantifiable benefits (e.g., prestige of the modern method, educational considerations) may play an equally important role.

**Fig. 1:**
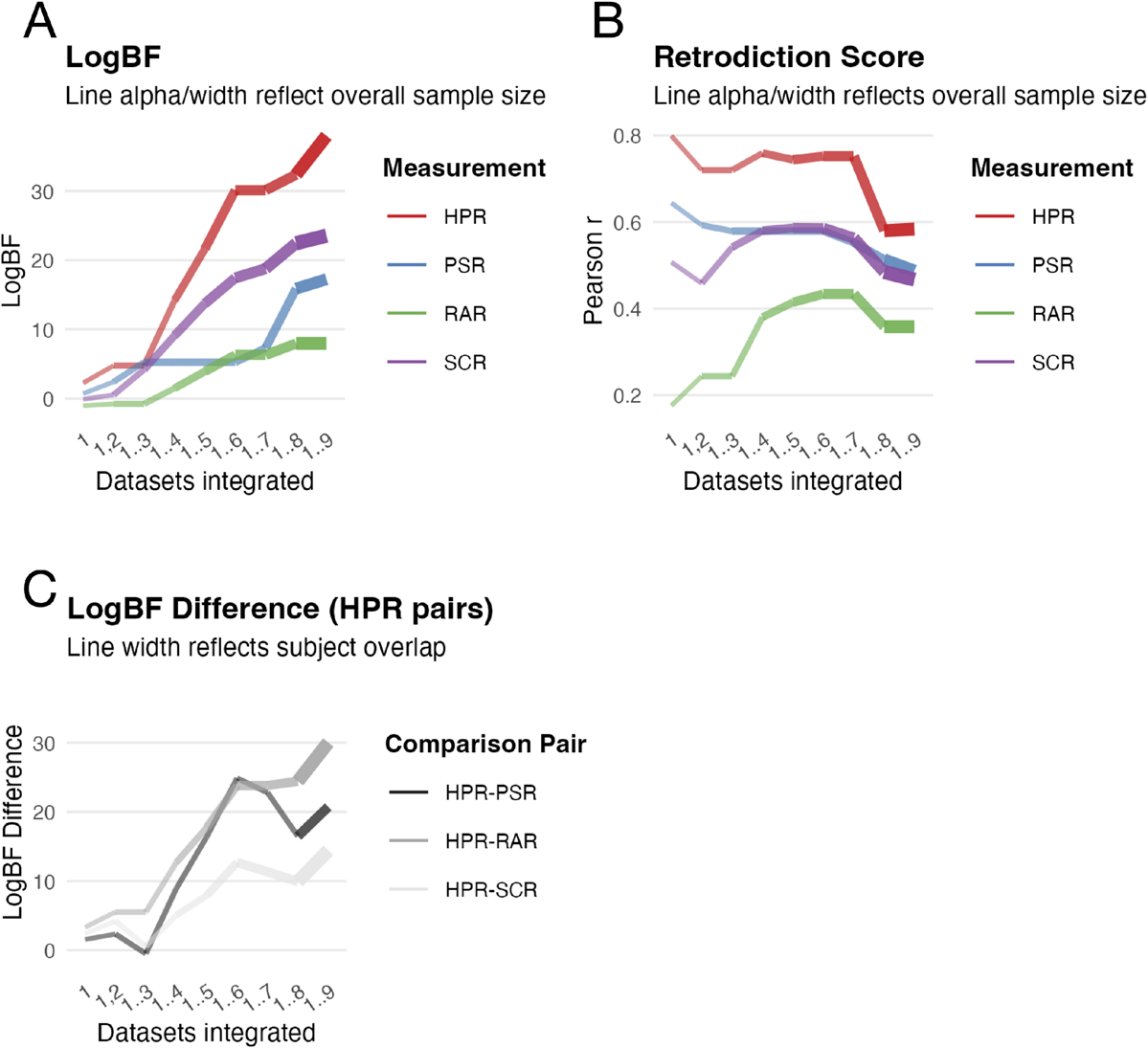
Comparison of the four measurement types examined in our study, across successive stages of dataset integration (ordered by acquisition date). **A**. Log(Bayes factor) for each measurement. **B**. Retrodictive validity scores, expressed as Pearson’s *r*, rather than the Cohen’s *d* values reported in Table 1. Both metrics are derived from *t*-statistics calculated on the accrued data. **C**. Differences in log Bayes factor between the highest-performing measure and the others, offering a focused comparison. Line width in C indicates the extent of subject overlap, serving as an index of the confidence in each comparison. In panels A and B, line width and alpha reflect the incremental sample size as datasets are accumulated. For clarity, the exact numbers of overlapping subjects are as follows: HPR-PSR: 114; HPR-RAR: 149; HPR-SCR: 175

In general, the sample size required in an experiment depends on the desired statistical power and the effect size. Because the relationship between effect size and required sample size is non-linear, even subtle increments in measurement accuracy can lead to substantial reductions in the required sample size.

To illustrate how to leverage calibration for power analysis, we focus on the common scenario of a two-group comparison in a substantive experiment. In this case, the effect size can easily be decomposed into effect magnitude and pooled standard deviation. For the case of Cohen’s *d*, we have:

4 $$\begin{aligned} d = \frac{\mu }{\sigma }, \end{aligned}$$

where $\mu$ is the effect magnitude, i.e., the average difference of the measured score between two groups, and $\sigma$ is the pooled standard deviation of the measured score. This variability depends on the measurement error, which we can assume is the same between the calibration experiment and substantive experiment, and the variability of the latent variable, i.e., experimental aberration. Because substantive experiments typically involve more heterogeneous conditions than calibration settings, we assume that the variability of the latent variable is equal to or greater than that in the calibration experiment. Hence, if $\sigma _c$ denotes the within-group standard deviation in the calibration experiment, we assume $\sigma _c \le \sigma$.

Now, researchers can specify the minimum effect magnitude of interest. One option is to directly specify the minimum effect magnitude $\mu _{min}$ on the scale of the measured score. Then inserting this into eq. (4) yields:

5 $$\begin{aligned} d = \frac{\mu _{min}}{\sigma } \le \frac{\mu _{min}}{\sigma _c}. \end{aligned}$$

To compute the right-hand side, *CalibR* reports the within-group standard deviation for each observable. Practically, when performing power analyses using *CalibR*, users can input an attenuation factor $\alpha$, with $0 < \alpha \le 1$, that encodes assumptions about increased variability in the substantive experiment relative to the calibration setting. Specifically, $\alpha = \sigma _c / \sigma$, so that

6 $$\begin{aligned} d = \alpha \frac{\mu _{\min }}{\sigma _c}. \end{aligned}$$

Values closer to 0 correspond to stronger variance inflation in the substantive experiment, whereas values closer to 1 reflect conditions similar to those of the calibration experiment.

A second scenario, frequently encountered in intervention research, is that a calibration experiment is “embedded” in the substantive experiment. Consider the case of pharmacological effects on fear conditioning. Here, an experiment frequently consists of two groups, both undergoing a standard fear conditioning procedure with two stimulus conditions: a conditioned stimulus paired with an aversive outcome (CS+, i.e., the reinforced cue) and a conditioned stimulus not paired with the outcome (CS−, i.e., the non-reinforced cue), with one group under placebo and the other under drug treatment. Here, we can assume that the effect size in the placebo group is the same as in a calibration experiment with two conditions (we could also posit that the effect is attenuated; in which case, an attenuation factor can be introduced). Now we can specify the minimum relevant between-group effect magnitude with respect to the effect magnitude in the placebo group. For example, we might stipulate that, for an effect to be considered meaningful, the difference in conditions within the treatment group must be at least 50% larger compared to the placebo group. Assuming homogeneity of standard deviations across groups, we can formalize this criterion mathematically. Denoting the condition-specific means with $\mu _{CS \pm }$, and the placebo and treatment groups with subscripts *p* and *t*, the standardized difference can be expressed as:

7 $$\begin{aligned} d= &  \frac{\mu }{\sigma } = \frac{(\mu _{t,CS+}-\mu _{t,CS-}) - (\mu _{p,CS+}-\mu _{p,CS-})}{\sigma }\nonumber \\= &  0.5\,\frac{(\mu _{p,CS+}-\mu _{p,CS-})}{\sigma } \le 0.5\,\frac{\mu _c}{\sigma _c} = 0.5\,d_c \, . \end{aligned}$$

where $\mu _c$ and $d_c$ denote the effect magnitude and effect size in the calibration experiment, respectively. To facilitate computing this effect size, *CalibR* further reports Cohen’s *d*, alongside Pearson’s *r*, for calibration experiments with just two standard values.

Once we have computed our desired effect size *d* for different measurement methods, we can multiply the difference in required sample size by the per-subject research costs and subtract any investments that need to be made to implement the better measurement method. The result then constitutes the economic benefit of the superior measurement method.

**Table 1:** Retrodictive validity (expressed here as Cohen’s *d*) for multiple fear conditioning datasets, assessed across four physiological measures: skin conductance response (SCR), pupil size response (PSR), respiration amplitude response (RAR), and heart period response (HPR). For each dataset, the sample size and date of data collection are reported. Missing measurement values are indicated by a dot ($\cdot$)

Dataset	Retrodictive validity (Cohen’s *d*)				Sample size	Date
	SCR	PSR	RAR	HPR		yyyy-mm
SC4B	0.75	1.08	0.22	0.77	8	2017-10
PubFe	0.44	0.93	0.35	0.74	12	2018-03
VC7B	0.77	0.66	$\cdot$	$\cdot$	17	2018-04
DoxMemP	0.81	$\cdot$	0.55	1.26	20	2018-07
FR	0.74	$\cdot$	0.52	1	22	2018-07
TC	0.70	$\cdot$	0.55	1.20	19	2018-07
FSS6B	0.44	0.49	$\cdot$	$\cdot$	17	2020-01
FER02	0.40	0.52	0.28	0.26	68	2020-03
FER01	0.40	0.37	$\cdot$	0.74	26	2021-11

## Worked example

To illustrate the use of the proposed methods and *CalibR*, we analyze datasets comprising multiple measurement modalities for fear conditioning. All data are publicly available (Mancinelli et al., 2024). For illustration purposes, we treat them as if only the retrodictive validity scores had been successively published for each experiment. This reflects a situation in which multiple calibration experiments are performed over extended periods of time. Notably, these experiments were not designed as calibration experiments, and so the experimental aberration might be larger and more heterogeneous between studies than in a dedicated calibration experiment. However, this is not expected to affect the ranking of methods.

In each of these experiments, a subset of four different measurement methods for quantification of fear conditioning is compared. These methods are based on conditioned skin conductance responses (SCR), pupil size responses (PSR), respiration amplitude responses (RAR), and heart period responses (HPR). Each experiment entails two conditions: a conditioned stimulus (CS+) predicting an electric shock (unconditioned stimulus, US), and another CS predicting the absence of the US. Because there are only two standard values, the retrodictive validity score (normally expressed as a Pearson correlation between standard and measured scores) can equivalently be written as Cohen’s d for the within-subject difference between the two conditions, which is the metric most commonly reported in the fear conditioning literature. In Fig. 1, we report retrodictive validity in terms of Pearson’s r to emphasize the general regression-based formulation of the calibration framework. Thus, Table 1 summarizes the measurement methods and their corresponding retrodictive validity in terms of Cohen’s *d* for each dataset, along with sample sizes and publication dates.

### Inference

We now address the question of which measurement method performs best, proceeding in an iterative fashion. Figure 1 shows the evolution of evidence (panel A) and retrodictive validity scores (panel B) across datasets; note that this figure expresses retrodictive validity in the more general form of Pearson’s *r*.

Because most experiments contain only a subset of measurement methods, evidence and retrodictive validity evolve in different ways. For example, the retrodictive validity score of PSR and SCR remains similar between datasets 4 and 7, but their evidence diverges, with more evidence for SCR than PSR. This indicates that at this stage, SCR and PSR continue to have similar retrodictive validity in the sample, but because there are more data for SCR, we can be more certain for SCR than for PSR that retrodictive validity is truly better than the baseline method. Similarly, although retrodictive validity is always higher for PSR than RAR, model evidence for RAR reaches the same level as for PSR for datasets 7-8, simply because there is (much) more data for RAR at this stage, and thus our confidence in the retrodictive validity score for RAR is (much) higher than for PSR. Thus, model evidence, integrated across datasets with some methods unavailable, combines the sample accuracy of a measurement method with our confidence that this sample accuracy corresponds to the true accuracy.

### Decision-making

Based on the model evidence and retrodictive validity alike, HPR emerges as the most accurate measurement method. To illustrate the practical implications, consider the scenario of transitioning from SCR to HPR to improve measurement in a given pre-registered experiment testing the impact of an intervention (e.g. a pharmacological compound) on fear acquisition. We assume that a substantively meaningful intervention effect corresponds to a reduction in fear acquisition of at least 50% relative to placebo. For power analysis, we work with the magnitude of this contrast, so that the relevant standardized effect size is given by the absolute value of the between-group difference. This results in a minimum relevant effect size of Cohen’s $d=.49$ for HPR and $d=.36$ for SCR (i.e. half of the original calibration effect size). Achieving a desired statistical power of 0.8 at a significance level of 0.05 in a one-tailed *t*-test would require a sample size, per group, of 52 when using HPR, compared to 96 for SCR. Thus, overall 88 fewer participants are required to achieve the same statistical power when basing inference on HPR rather than SCR. Assuming a per-participant cost of 250 € for a drug study (work time, pharmacy cost, participant reimbursement, lab cost), using HPR would entail savings of 22,000 €. This needs to be offset against the switch cost, e.g. for buying HPR equipment and training staff accordingly.

## Discussion

In this paper, we addressed the practical challenges of interpreting, and combining, retrodictive validity scores in the context of experiment-based calibration, a framework designed to compare the measurement accuracy of multiple measurement methods. Unlike traditional psychometric approaches reliant on individual differences and requiring multi-trait multi-method matrices (Bach et al., 2025), calibration exploits experimentally manipulated latent variables, providing a formal framework for assessing measurement validity.

We addressed two crucial matters in a calibration setting: inference, and decision-making. These aspects become significantly complex due to the potentially large and initially unknown number of measurement methods, and the likely chance that some methods may not be available across all datasets.

The proposed approach leverages Bayesian model comparison, which is uniquely suited to handle multiple simultaneous comparisons of measurement methods, effectively mitigating the limitations inherent in classical null hypothesis significance testing. Bayesian model comparison provides an explicit probabilistic framework to quantify evidence for the relative superiority of measurement methods, incorporating both the likelihood of the data under each model and prior beliefs (or prior information available).

As a measure of retrodictive validity, we used the (log) Bayes factor for the retrodiction model of a measurement, against that of a null measurement which is completely non-retrodictive (a constant measurement that is identical across standard scores). The model-evidence for this null measurement functions practically as a “unit of measure” for each dataset. The evidence is integrated across datasets in a meta-analytic fashion as outlined in Nikolakopoulos and Ntzoufras (2021).

An important limitation of our approach is that Bayes factors are conditioned on the specific data to which the models are fitted. Accordingly, calibration is fully sound when all measurement methods subject to comparison are evaluated against identical underlying datasets. In practice however, studies sometimes include only subsets of methods, so the effective data overlap follows from measurement availability rather than shared experimental design (this happens in our worked example above, see Table 1). *CalibR* explicitly reports the method-induced overlap across studies, and gives the option to restrict comparisons to fully overlapping datasets. When comparing log Bayes factors across non-overlapping datasets, the degree of overlap can be treated as an indicator of comparison reliability (Fig. 1C); ideally, conclusions should be checked for stability under overlap-restricted analyses.

Our framework for combining evidence across studies is akin to hierarchical meta-analysis. *CalibR* implements a fixed-effect synthesis of study-level retrodiction statistics, which is appropriate when calibration datasets are sufficiently similar to support a common interpretation of the accumulated evidence. A random-effects model would relax this assumption, but established frameworks typically treat between-study variation as Gaussian noise. This is appropriate when heterogeneity is unstructured, but less so when differences between datasets are systematic - for example, when method availability is linked to study size, acquisition period, or other design features, as is often the case in calibration data. Because our fixed-effects approach makes fewer parametric assumptions, it appears more parsimonious. Furthermore, in practical settings, misspecified random effects models tend to be difficult to estimate, with results sometimes depending on the particular approximations used. Future work could extend our framework to include hierarchical or meta-regression approaches that model structured heterogeneity explicitly.

Our inferential approach is implemented within the software package *CalibR*. The package is designed to be user-friendly and generalizable, facilitating integration and interpretation of calibration results across multiple datasets.

In our worked example, using real data from Mancinelli et al. (2024), we calibrated fear conditioning measures across multiple datasets. The main result is encapsulated in panel C of Fig. 1, as HPR demonstrates the highest retrodictive validity. However, as mentioned, overlap among datasets also requires attention. For instance, overlap with PSR is the lowest, suggesting results may be more susceptible to change – whereas overlap with SCR is the highest, indicating greater reliability. Nevertheless, we can conclude that HPR represents the most accurate method for assessing fear conditioning according to these data (Mancinelli et al., 2024).

The inferential framework included in *CalibR* supports optimal decision-making by quantifying the immediate economic benefits achieved through adopting superior measurement methods. This is operationalized by calculating the potential reduction in required sample sizes resulting from improved measurement accuracy. Such reductions have clear practical and financial advantages, allowing analysis and quantification, for instance, at the pre-registration stage, of the investment into new resources or methodological refinement.

In conclusion, by integrating Bayesian model comparison and decision-theoretic analysis within the *CalibR* software, this paper significantly enhances the inferential methodology, and thus practical utility, of experiment-based calibration. It provides a robust methodological framework for measurement validation, well-suited to the complexities encountered in psychological research and beyond.

## Data Availability

All data are available on OSF, at https://osf.io/cmaq7/.

## Code availability

*CalibR* is available at https://github.com/bachlab/calibr.
